# Effects of graphene oxide on PCR amplification for microbial community survey

**DOI:** 10.1186/s12866-020-01965-7

**Published:** 2020-09-11

**Authors:** Shuzhen Li, Zhujun Wang, Yuanyuan Wang, Maoyong Song, Guangxin Lu, Ning Dang, Huaqun Yin, Yuanyuan Qu, Ye Deng

**Affiliations:** 1 CAS Key Laboratory of Environmental Biotechnology, Research Center for Eco-Environmental Sciences, Chinese Academy of Sciences, Beijing, 100085 China; 2 Key Laboratory of Industrial Ecology and Environmental Engineering (Ministry of Education), School of Environmental Science and Technology, Dalian University of Technolog, Dalian, 116024 China; 3 College of Resources and Environment, University of Chinese Academy of Sciences, Beijing, 100049 China; 4 State Key Laboratory of Environmental Chemistry and Ecotoxicology, Research Center for Eco-Environmental Sciences, Chinese Academy of Sciences, Beijing, 100085 China; 5 College of Agriculture and Animal Husbandry, Qinghai University, Xining, 810016 China; 6 School of Minerals Processing and Bioengineering, Central South University, Changsha, 410083 China

**Keywords:** Oxidized graphene, Bacteria, Fungi, Chimera, Community composition

## Abstract

**Background:**

Graphene oxide (GO) has been suggested as an efficient assistant additive to eliminate non-specific amplification of the polymerase chain reaction (PCR). Although many studies have focused on exploring its molecular mechanism, the practice of GO on the quantitation of microbial community has not been implemented yet. In this study, GO was added in PCR system to explore the changes on removing typical amplification errors, such as chimera and mismatches on two kinds of mock communities (an evenly mixed and a staggered mock communities) and environmental samples.

**Results:**

High-throughput sequencing of bacterial and fungal communities, based on 16S rRNA genes and internal transcribed spacers (ITS) respectively, showed that GO could significantly increase large segmental error (chimeric sequence) in PCR procedure while had no specific effect on point error (mismatched sequence). Besides, GO reduced the α-diversity of community, and changed the composition of fungal community more obviously than bacterial community.

**Conclusions:**

Our study provides the first quantitative data on microbial community level to prove the negative effect of GO, and also indicates that there may be a more complex interaction between GO and comprehensive DNA fragments in PCR process.

## Background

In recent years, the study of environmental microbiome is undergoing a great revolution by the development of next-generation sequencing approaches and the establishment of robust bioinformatic tools [1, 2]. The sequential steps of conducting a microbiome study have been systematic and comprehensive raised by researchers [3]. After preliminary works finished, for example, sample collection and DNA extraction, PCR-based marker gene survey methods are applied. A segment of a conserved sequence such as the 16S ribosomal RNA (rRNA) gene for bacteria, or internal transcribed spacers (ITS) region for fungi, is amplified and sequenced, to quantify and visualize the microbial community composition, distribution and diversity that made up by operational taxonomic units (OTUs).

It has been a common consent that PCR has become one of the most ubiquitous and important tools in molecular biology since it was developed in 1985 [4]. However, the amplification efficiency of PCR often decreased with the production of non-specific DNA fragments, especially in multiple-round PCR. Till now, many factors have been found to affect the specificity of PCR, such as chimeric reads, primer mismatches or amplification mismatches and sequencing errors, which are frequently included in the PCR mixture [5–9]. Usually, when the concentration of template DNA is very low, or the structure of DNA template is very complicated, such as GC-rich gene or mammalian genomic DNA, the specificity of PCR might be very low [10].

Graphene, with its huge surface area, has excellent electronic conductivity, heat transfer and mechanical strength properties, which make it a remarkable candidate for biological applications [11]. Graphene oxide (GO) is an oxidized form of graphene, incorporated with oxygen-containing groups on the surface, such as epoxy, hydroxyl and carboxyl groups, resulting in high polarity and hydrophilicity [12, 13]. Recently, Graphene oxide has been suggested as an efficient assistant additive to eliminate non-specific amplification of the polymerase chain reaction [14, 15]. Some mechanisms have been proposed so far are as follows. (1) Enhanced thermal conductivity [16]. The enhanced heat transfer effect of nanoparticles has been a widely accepted notion. The presence of GO in PCR amplification helps a better dissipation of heat in all denaturation, annealing and extension steps, which makes these processes more rapidly. Good heat dissipation may be due to collision among GO, base fluid molecules, and PCR reagents. Reaction components aggregated around GO increase the efficient of dynamical contact among reaction components, hence it may result in heat equilibrium in the reaction and enhance PCR efficiency. (2) Interaction of DNA polymerase with GO [10, 17]. As GO surface is negatively charged, the adsorbed amount of positively charged *Pfu* polymerase and Mg^2+^ is relatively high at low GO concentration. As a result, PCR reagents such as dNTPs, DNA template and primers with negatively charged are attracted by positively charged *Pfu* polymerase on GO surface. Thus, the probability of mismatch is decreased and the specificity of PCR is improved. (3) Binding of DNA and GO [18, 19]. PCR reagents such as primers and single-stranded DNA can selectively stack to GO, which prevents their self-folding and thus improves the sensitivity and specificity of PCR by enhancing the efficiency of the base-pairing between the primers and template. Although many studies have been focusing on exploring the influence of GO on PCR procedure by electrophoresis, what the specific effects by the addition of GO would have on the number of typical amplification errors, such as chimeras and mismatches, has not been systematically studied. Besides, whether GO will improve the PCR performance for the survey of microbial community, such as the influences on community composition and diversity, has not been explored yet.

Here in this study we explore the effect of GO on removing amplification errors, including chimeric and mismatched sequences on two kinds of mock communities (an even and a staggered mock communities) and environmental samples, as well as its contribution on the changes of community diversity and composition.

## Results

### GO affects amplification errors in mock communities

**Fig. 1:**
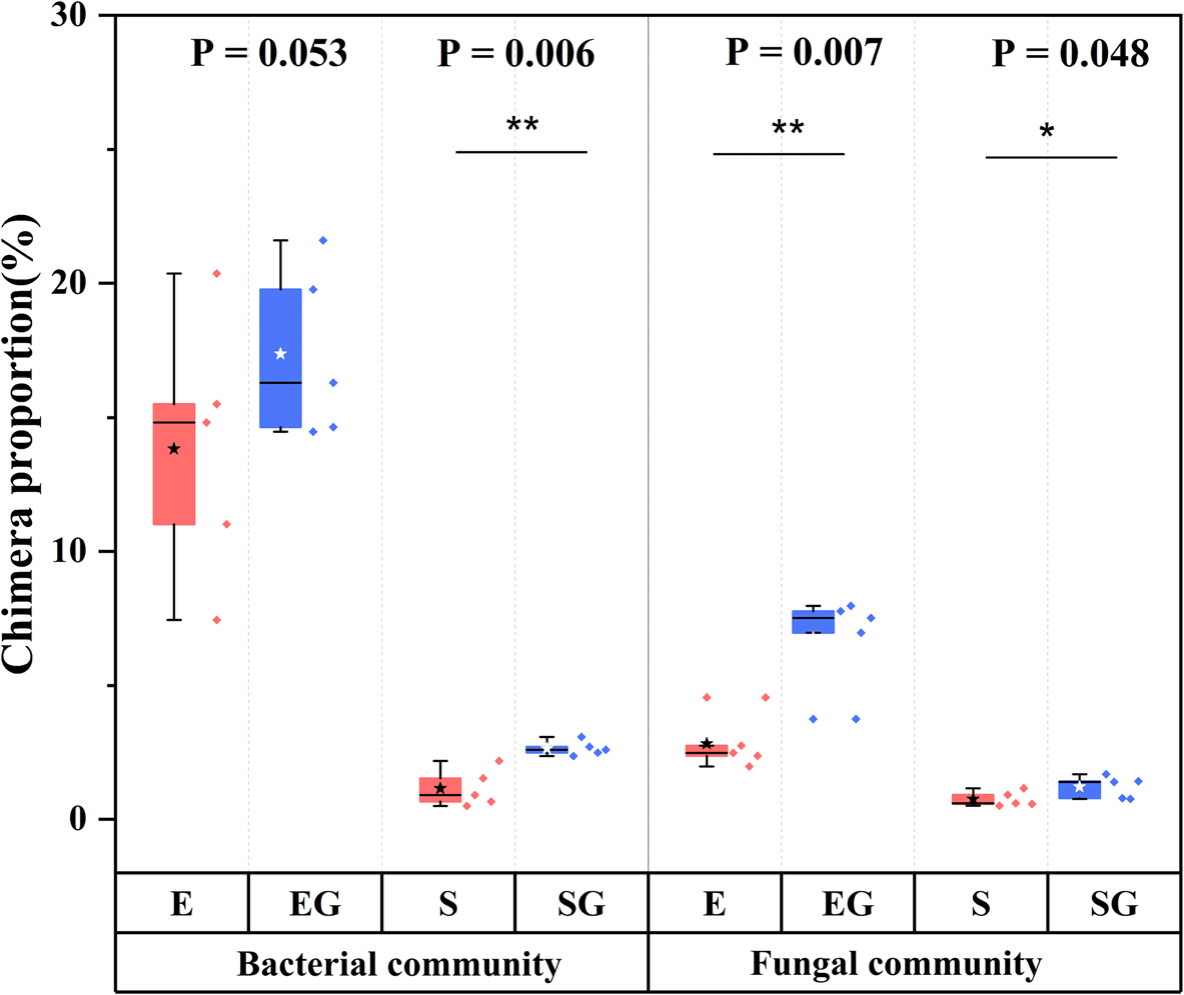
Detected chimera proportions in mock communities. E, even mock community; EG, even mock community with GO; S, staggered mock community; SG, staggered mock community with GO. Star in boxplot is the average value

**Fig. 2:**
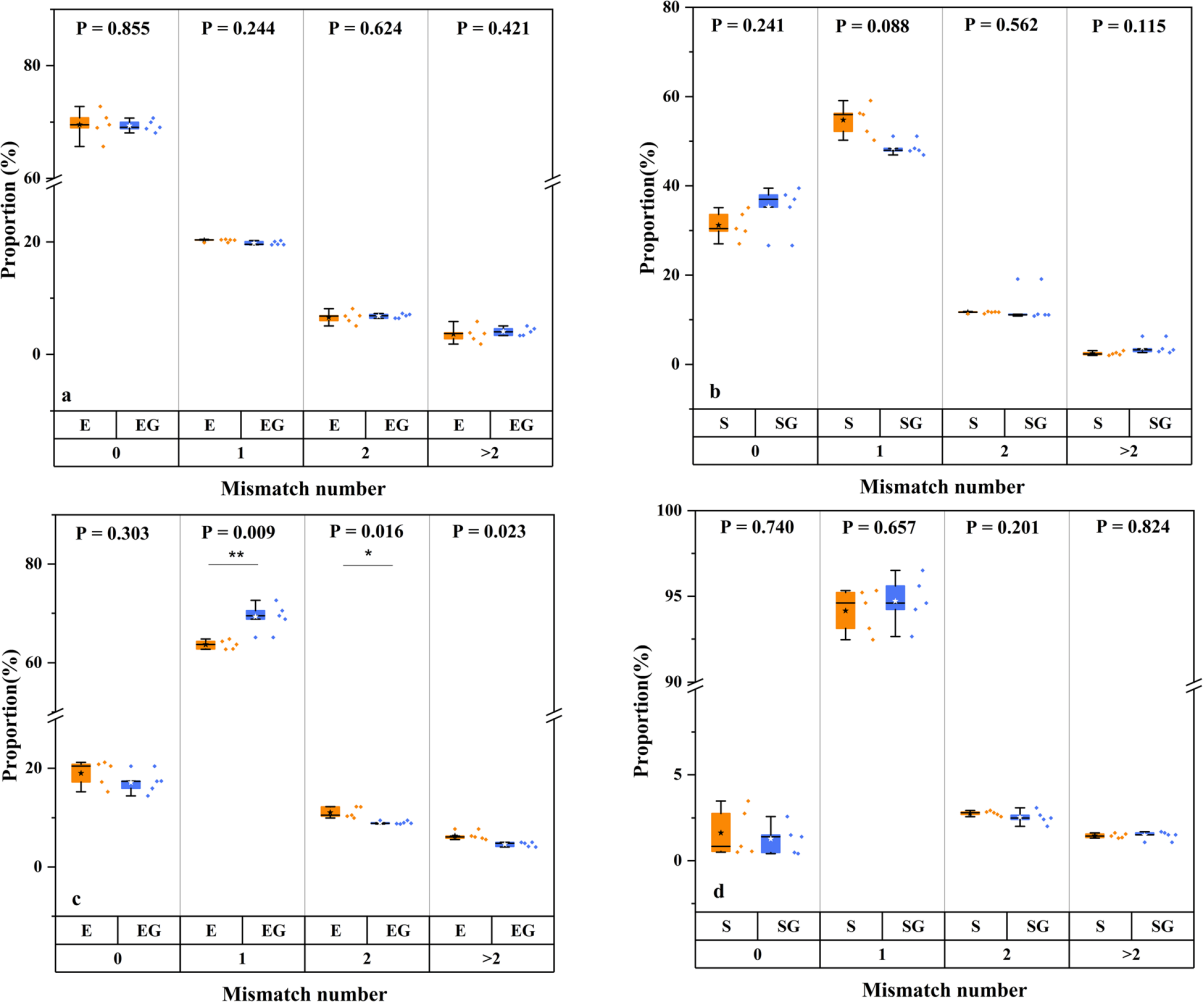
Mismatched sequences. **a** even bacterial mock community; (**b**) staggered bacterial mock community; (**c**) even fungal mock community (**d**) staggered fungal mock community. Star in boxplot is the average value

Sequences of mock communities were filtered based on quality score, and retained sequences were firstly used to explore the influence of GO on chimeras and mismatches. As shown in Fig. 1, the addition of GO in PCR showed a great effect on chimera formation. More chimera generated in GO groups, especially for staggered bacterial mock community (*P* = 0.006), as well as evenly mixed (*P* = 0.007) and staggered (*P* = 0.048) fungal mock communities. Bacterial community generated a larger proportion of chimeric sequences than fungal community in both even and staggered mock communities, and it seemed that chimera proportion increased more significantly in fungi than in bacteria after the addition of GO (Fig. 1). Instead, we observed that GO showed litter impact on mismatch (Fig. 2). In any kinds of the mock communities of bacteria and fungi, there was no significant difference between control and GO groups in most of the mismatch numbers (*P* > 0.05), except for one and two mismatches in even fungal mock community.

### GO changes the composition of environmental communities

**Fig. 3:**
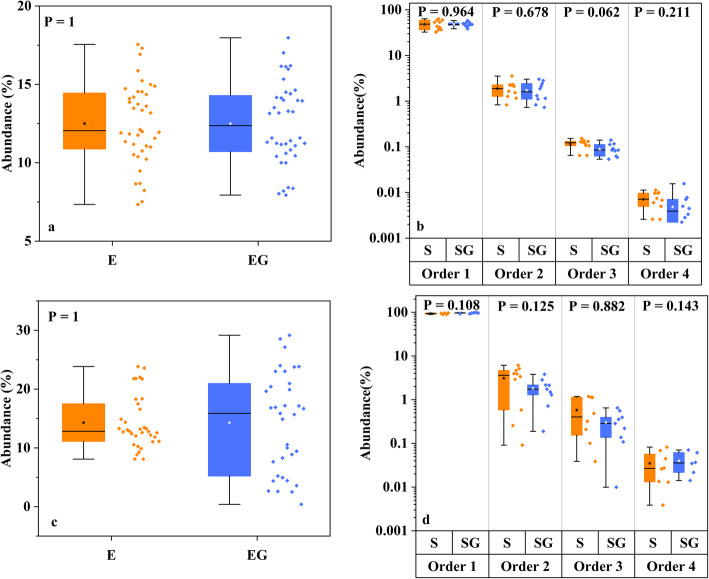
Even and staggered mock community mean abundance. **a** even bacterial mock community; (**b**) staggered bacterial mock community; (**c**) even fungal mock community (**d**) staggered fungal mock community. Star in boxplot is the average value

The next important question is whether GO can change the composition of microbial community. After removing chimeric sequences, OTUs were generated and we compared the abundance of samples in control and GO groups in mock community. Results showed that there was no significant increase or decrease in relative abundance in both even and staggered mock communities for bacteria and fungi (*P* > 0.05) (Fig. 3).

**Fig. 4:**
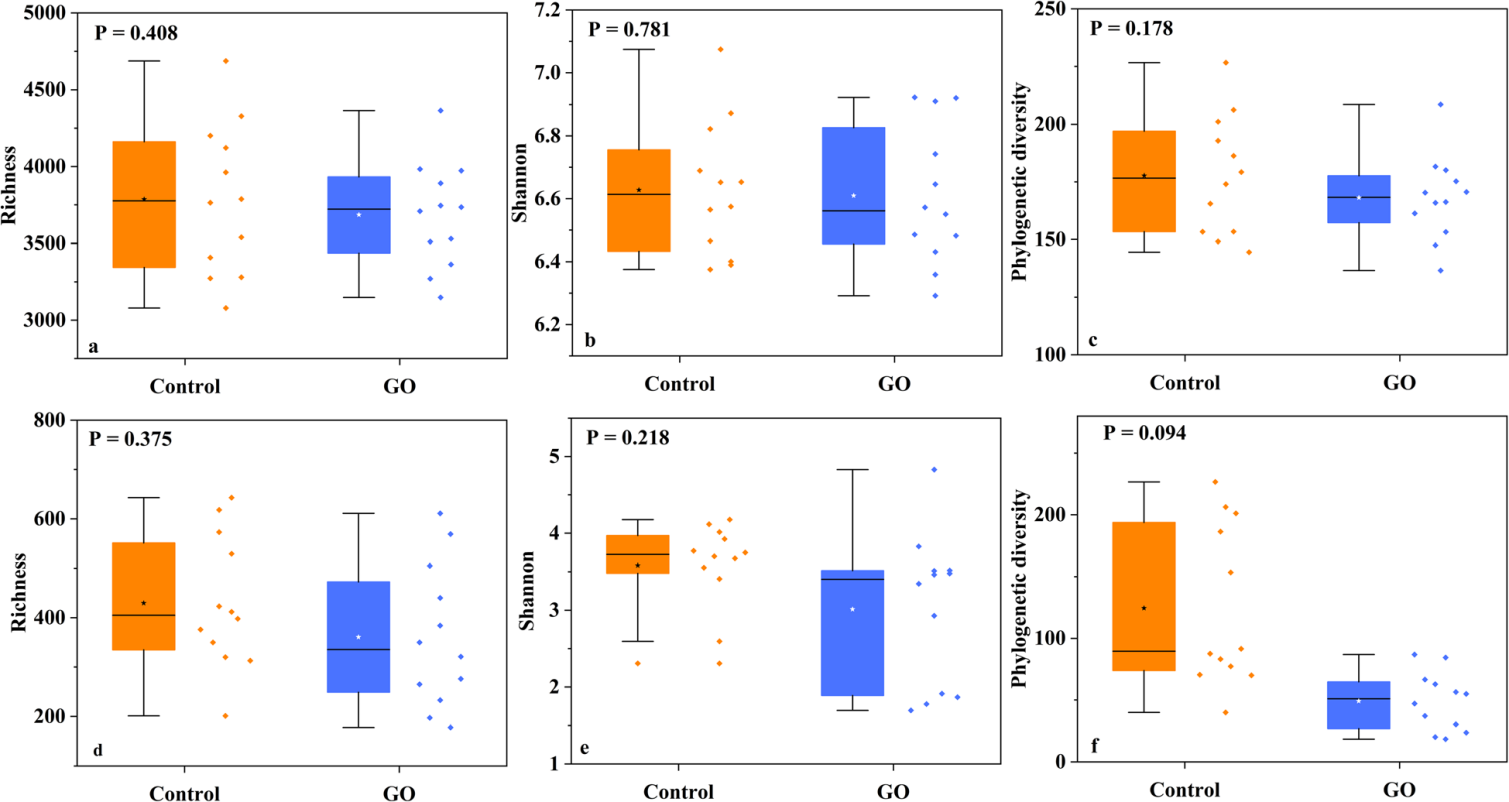
Alpha diversity indexes (Richness, Shannon and Phylogenetic diversity) for environmental samples. **a**-**c** bacteria data; **d**-**f** fungi data. Star in boxplot is the average value

**Fig. 5:**
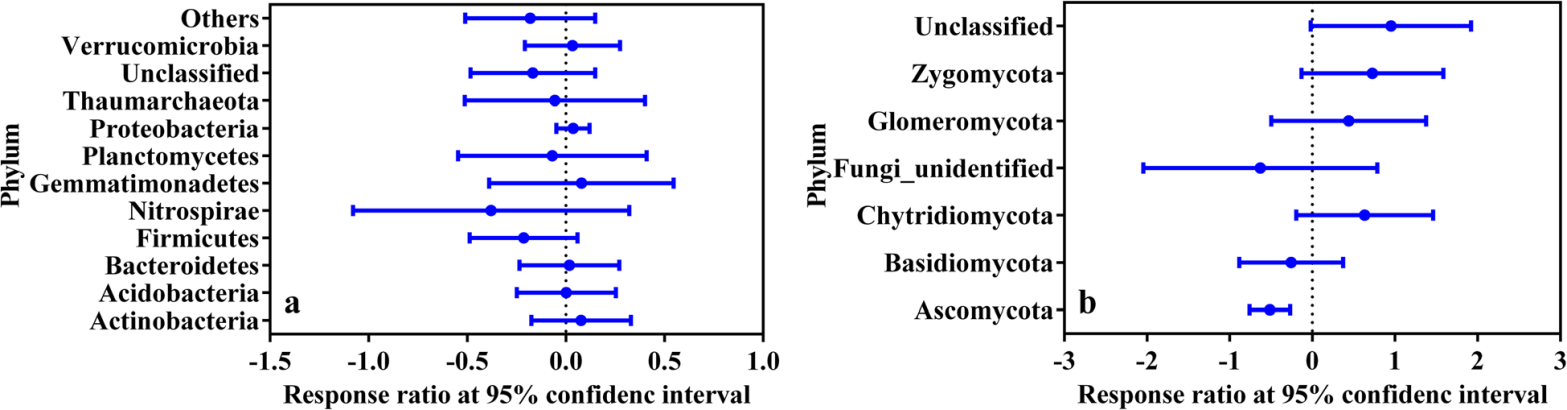
Response ratio analysis of environmental community in phylum level. **a** bacteria data; **b** fungi data

To further validate the results and reduce deviations from the mock community, 24 soil samples were collected. After all quality control process, a total of 1,922,267 and 849,199 sequences were obtained for bacterial and fungal communities, respectively (Table S1). OTUs were generated and the average sequence numbers of each sample were 53,300 and 33,846 for bacterial and fungal communities, respectively (Table S2). Samples were randomly sub-sampled to an equal depth, and three kinds of α-diversity indexes, including richness, Shannon index and phylogenetic diversity were measured. Results showed that the addition of GO reduced α-diversity in bacterial and fungal communities (Fig. 4, Table S3). We further assessed taxonomic differences on phylum level between control and GO groups in environmental samples by response ratio analysis [20]. After replacing ddH_2_O to GO in PCR protocol, bacterial communities did not show a significant change (Fig. 5a). Abundances of Actinobacteria and Gemmatimonadetes had a slight rise, abundances of Firmicutes and Planctomycetes decreased, and other main phyla remained almost unchanged. In fungal communities (Fig. 5b), abundance of Ascomycota had a significant decrease. Basidiomycota also showed a decreased abundance, and abundances of Zygomycota, Glomeromycota, and Chytridiomycota went up, while the differences were no significant between control and GO groups.

## Discussion

### Effect of GO on PCR amplification and microbial community

Chimeras and mismatches are two kinds of typical amplification errors, which are easy to appear in PCR process and greatly reduce its specificity. Chimeras are hybrid products formed from two or more biological sequences during PCR process. The most common mechanism is believed to be incomplete template extension in which arise most chimeras. When the sequences are amplified in conserved regions, such as bacterial 16S rRNA or fungal ITS region, chimera is more likely to occur and further bring biases to downstream analysis [21]. Besides, in the dozens of cycles of PCR amplification, it is impossible to ensure that any points of a single sequence do not mismatch. Every mismatch will cause non-specific amplification in the next PCR cycle. Hence, it is very important to examine whether GO has an effect on the occurrence of chimeras and mismatches.

From our results, it could be speculated that GO affected large segmental errors during amplification, that is, significantly increased the number of chimeric sequences (Fig. 1). Meanwhile, GO showed no obvious effect on point errors of single or multiple nucleotides, i.e. mismatched sequences (Fig. 2). Although previous studies have demonstrated that GO can act as an efficient assistant additive to eliminate non-specific amplification [10, 14], our study provides the quantitative data on microbial community level to prove the negative effect of GO. On the other hand, these observations are based on our mock communities. The mock community has been demonstrated to be a good positive control with a known set of qualitative and quantitative compositions to explore the dynamic changes of microbial diversity and composition [22, 23]. The relatively small number of species used to construct mock communities may limit the further generalization of our results. Nonetheless, our results were enough to indicate that GO will not promote PCR for all microbial communities, on the contrary, GO can perform adverse effects on PCR efficiency for some bacterial and fungal taxa.

After incorporating environmental samples into the study, we found that there was a dynamic change of community diversity and composition (Figs. 4, 5). We proposed two possible reasons for community changes. Firstly, according to previous studies, the addition of GO effectively reduce non-specific amplification [14, 15]. Based on this we can speculate that GO may reduce the OTU inflation, which leads to more accurate community structure. Secondly, based on the results of our previous mock community, GO may have made some species more prone to generate incomplete amplification, thus more chimera appeared. More chimeric sequences for some taxa are removed during the quality control process, resulting in decreases in diversity and variations in composition of microbial community.

### The effect of GO on DNA may be more complicated than expected

Previous studies have proved that GO can eliminate non-specific amplification effectively [14, 15]. However, their conclusions and proposed mechanisms lack quantitative data at the community level. Meanwhile, interactions between GO and DNA have been widely studied in many fields, and researches have indicated that GO has strong toxicity to DNA through the destructive effect [24]. Besides, the double helix structure of DNA could be disrupted by graphene-based nanoparticles through strong dispersive forces [25]. More generally, other studies have been focusing on the interactions between various metal oxide nanoparticles materials and DNA amplification, and both of inhibition and enhancement effects have been observed extensively in independent researches. PCR experiments have given evidence that multiple nanoparticles (e.g. ZnO, CeO_2_, citrate-stabilized AuNPs and AgNPs) can inhibit DNA amplification [26]. Nanoparticles can bind to DNA to change the normal conformation of DNA molecules, and this high affinity can further inhibit the functions of DNA polymerases [27–29]. On the contrary, some types of nanoparticles (e.g. Fe_3_O_4_NPs and colloidal AuNPs) have the ability to promote PCR specificity, efficiency or yield [30–33]. Considering all knowledge, the influence of GO on DNA may be a combination of promoting and toxic effects. In the complex PCR mixture, GO enhances thermal conductivity, binds DNA and interacts with DNA through electrical charge, these can improve the specificity of PCR. But at the same time, GO also performs destructive or inhibitory functions as a typical graphene-based nanoparticle. Moreover, the impact of GO may have species-specific effect, different types of microbes may have differential responses under GO exposure [34]. GO can modify and shape the microbial community structure through inducing inhibition or promotion of particular species’ DNA replication, which may account for the community changes [34–37].

## Conclusions

The results presented in this study represent important contributions to understand the effect of GO on the generation of chimeric and mismatched sequences, and the change of composition and diversity of microbial communities. GO significantly increases chimeric sequences but shows no specific effect on mismatch. Besides, GO reduces the alpha diversity of environmental community, and changes community composition more obviously in fungal community than in bacterial community. Our study provides the first quantitative data on microbial community to prove the negative effect of GO, and proposes that there may be a more complex interaction between GO and DNA in PCR process. Our research makes a preliminary exploration on community level, and future studies are needed to take a closer look at the role of GO and the mechanisms behind it.

## Methods

### Construction of mock community

Bacterial and fungal mock community were constructed by eight different species, respectively. For bacterial mock community, species derived from eight different genera, including *Alcaligenes* sp. (Accession number JF698681), *Arthrobacter* sp. (Accession number FJ851358), *Bacillus* sp. (Accession number KU556329), *Cupriavidus* sp. (Accession number KU726429), *Patulibacter* sp. (Accession number KT581436), *Pseudomonas* sp. (Accession number NZ_AHGZ00000000), *Terrimonas* sp. (Accession number NZ_AUDS01000000) and *Arthrobacter* sp. (Accession number NZ_JWMD01000000). For fungal mock community, species were obtained from the Agricultural Culture Collection of China (ACCC), that is *Auricularia auricula* (ACCC number 51049), *Cordyceps militaris* (ACCC number 50985), *Lentinula edodes* (ACCC number 50749), *Alternaria alternate* (ACCC number 38066), *Mucor racemosus* (ACCC number 30522), *Trichoderma reesei* (ACCC number 30590), *Fusarium oxysporum* (ACCC number 37404) and *Yarrowia lipolytical* (ACCC number 20101). Then genomic DNA of each species was extracted using TIANamp DNA Kit (Tiangen biotech Co Ltd.). Full 16S rRNA gene and ITS region were amplified by pair-wise universal primer 27F (5′-AGAGTTTGATCMTGGCTCAG-3′), 1492R (5′-GGYTACCTTGTTACGACTT-3′) and ITS1F (5′-CTTGGTCATTTAGAGGAAGTAA-3′), ITS4 (5′-TCCTCCGCTTATTGATATGC-3′), respectively [22, 38, 39]. Next, PCR products were purified and ligated with pMD18-T vector. The recombinant plasmid was cloned into *E. coli* DH5α, and DNA was extracted by TIANpure Mini Plasmid Kit (Tiangen biotech Co Ltd.). Two kinds of mock communities were constructed with different rRNA operon counts. Specifically, even mock community consisted of eight species at equimolar rRNA operon counts (5 ng/μL). Staggered mock community consisted of the same species with four gradients of rRNA operon counts (50, 5, 0.5 and 0.05 ng/μL). Each type of mock community made five biological replicates to ensure the robustness of the results.

### Environmental samples

Environmental microbial community samples were obtained from alpine meadow ecosystem in Qinghai province (33°24′30″N, 97°18′00″E) with an elevation of 4270 m. Samples taken from field station belongs to the typical plateau continental climate with a mean annual rainfall of 562.2 mm and a mean annual temperate of − 5.6 °C ~ 3.8 °C [40]. Twelve soil samples were collected from the depth of 15–30 cm. DNA was then extracted with FastDNA™ SPIN Kit for Soil (MP Biomedicals). Both of bacteria and fungi communities were amplified as follows.

### PCR, library preparation and high-throughput sequencing

For bacteria, V4 region of 16S rRNA gene was amplified using primers 515F (5′-GTGCCAGCMGCCGCGGTAA-3′) and 806R (5′-GGACTACHVGGGTWTCTAAT-3′) [41]. For fungi, ITS2 region was amplified by gITS7 (5′-GTGARTCATCGARTCTTTG-3′) and ITS4 (5′-TCCTCCGCTTATTGATATGC-3′) [22]. Both forward and reverse primers contained 12 unique base pair barcodes to distinguish samples. PCR conditions and library preparation are consistent with previous studies [42, 43]. Briefly, PCR mix contains 1 μL of template DNA within 20–30 ng/μL, 0.5 μL Taq DNA Enzyme, 1.5 μL dNTP mixture, 5 μL 10 × PCR buffer, 1.5 μL of both 10 μM forward and reverse primers and 39 μL ddH_2_O. The thermal cycle parameters were as follows: denaturation at 94 °C for 1 min, 30 cycles of 94 °C for 20 s, 57 °C for 25 s, 68 °C for 45 s, a final extension at 68 °C for 10 min and finally keep at 4 °C. As for exploring GO’s effect on community composition and diversity, 1 μg/mL GO has been showed with the greatest enhancement in PCR, and ddH_2_O was replaced by 1 μg/mL Go solution in our PCR system [14]. PCR products were separated by agarose gel electrophoresis and purified by Gel Extraction Kit (D2500–02, OMEGA BioTek). The purified DNA was quantified through NanoDrop 2000 Spectrophotometer (ThermoFisher, USA). All purified DNA were pooled together to construct a sequencing library and connect Illumina adapters directed by the protocol of VAHTSTM Nano DNA Library Prep Kit for Illumina® (Vazyme Biotech Co., Ltd) and MiSeq Reagent Kit Preparation Guide (Illumina). Pooled libraries were quantified using Qubit assay with Qubit 2.0 Fluorometer (Life Technologies). Sequencing was performed on an Illumina Miseq platform with 2 × 250 bp high-output run chemistry at Central South University, China.

### Quality control and bioinformatics approaches

Data from sequencing was analyzed by a publicly accessible pipeline (http://mem.rcees.ac.cn:8080) [44, 45]. Briefly, primers were removed and paired-end reads were joined by FLASH [46]. The minimum required overlap was 30 bp. Low-quality sequences were discarded with the threshold of Quality Score > 20, minimum length 140 and window size 5 by Btrim program [47]. Sequences length in 245–260 bp for bacteria, 240–320 bp for fungi were retained for further analysis. Then, chimeras were identified and removed by different reference databases for mock and environmental communities by UCHIME algorithm [48]. For bacterial and fungal mock communities, reference databases were eight bacterial or fungal sequences that have been sequenced to build the mock community. Sequences were detected as one of the targeted species by matching up to 97% sequence identity and 90% coverage by BLAST [49]. Meanwhile, mismatch was also identified according to BLAST outputs. For environmental community, reference databases for bacterial and fungal data were Greengene 13.8 taxonomy file and Gold database, respectively. Finally, high-quality clean sequences were classified into operational taxonomic units (OTUs) at 97% identity by UPARSE without any singletons being discarded [50].

For environmental samples, representative sequences were classified into different taxonomy by Bayesian classifier against the RDP training set and UNITE database for bacteria and fungi, respectively [51, 52]. According to sequences numbers, randomly sub-sampled OTU tables were generated to normalize total reads by 24,300 and 15,000 for bacterial and fungal samples. Alpha diversity indexes, including richness, Shannon and phylogenetic diversity were measured using vegan package in R (v.3.6.0) [53]. The significances between control and GO added groups were determined by independent and paired Student’s t-test as appropriate.

## Supplementary information

**Additional file 1.**

## Data Availability

The 16S rRNA gene and ITS region sequencing data analyzed in the current study are available in the NCBI Sequence Read Archive (SRA) database (https://www.ncbi.nlm.nih.gov/sra) under the accession numbers SUB7253148, SUB7257294, SUB7257355 and SUB7257383.

## Abbreviations

GO: Graphene oxide
PCR: Polymerase chain reaction
rRNA: Ribosomal RNA
ITS: Internal transcribed spacers
OTUs: Operational taxonomic units
