# Supplementary material for: Effects of graphene oxide on PCR amplification for microbial community survey
Source: BMC Microbiol. 2020 Sep 11;20:278. doi: 10.1186/s12866-020-01965-7 (PMC7488489; doi:10.1186/s12866-020-01965-7)
Supplement: Supplementary file 1 — Additional file 1. [file 12866_2020_1965_MOESM1_ESM.docx]

**Supplementary material**

**Effects of graphene oxide on PCR amplification for microbial community survey**

Shuzhen Li^1,2^, Zhujun Wang^1,3^, Yuanyuan Wang^4^, Maoyong Song^4^, [Guangxin Lu](https://www.sciencedirect.com/science/article/pii/S0038071719301968#!)^5^, Ning Dang^5^, Huaqun Yin^6^, Yuanyuan Qu^2^, Ye Deng^1,3,*^

1. CAS Key Laboratory of Environmental Biotechnology, Research Center for Eco-Environmental Sciences, Chinese Academy of Sciences, Beijing 100085, China

2. Key Laboratory of Industrial Ecology and Environmental Engineering (Ministry of Education), School of Environmental Science and Technology, Dalian University of Technology, Dalian, Liaoning 116024, China

3. College of Resources and Environment, University of Chinese Academy of Sciences, Beijing 100049, China

4. State Key Laboratory of Environmental Chemistry and Ecotoxicology, Research Center for Eco-Environmental Sciences, Chinese Academy of Sciences, Beijing 100085, China

5. College of Agriculture and Animal Husbandry, Qinghai University, Xining 810016, China

6. School of Minerals Processing and Bioengineering, Central South University, Changsha 410083, China

* Corresponding authors. E-mail: [yedeng@rcees.ac.cn](mailto:yedeng@rcees.ac.cn)

Table S1 Number of the reads in each step of data analysis.

| Data processing | Bacterial community | Fungal community |
| --- | --- | --- |
| Raw data | 2,265,857 | 1,172,756 |
| Flash | 2,215,922 | 1,117,905 |
| Btrim | 2,009,692 | 950,869 |
| Retained sequences for OTU generation | 1,922,267 | 849,199 |

Table S2 Reads per sample after OTU generation.

| Sample | Bacterial community | Fungal community |
| --- | --- | --- |
| A1 | 14,571 | 32,016 |
| A3 | 47,563 | 19,254 |
| E1 | 28,194 | 37,323 |
| E3 | 25,115 | 67,108 |
| F1 | 47,452 | 45,288 |
| F3 | 24,305 | 40,545 |
| H1 | 45,806 | 37,331 |
| H3 | 32,945 | 31,692 |
| L1 | 57,784 | 35,137 |
| L3 | 28,939 | 5,676 |
| M1 | 105,039 | 17,265 |
| M2 | 34,227 | 8,529 |
| A1_GO | 26,760 | 35,856 |
| A3_GO | 42,849 | 57,122 |
| E1_GO | 48,025 | 32,671 |
| E3_GO | 36,283 | 43,899 |
| F1_GO | 51,692 | 79,848 |
| F3_GO | 104,895 | 19,021 |
| H1_GO | 99,932 | 46,594 |
| H3_GO | 79,264 | 17,056 |
| L1_GO | 125,207 | 34,557 |
| L3_GO | 42,896 | 18,285 |
| M1_GO | 46,430 | 35,069 |
| M2_GO | 83,010 | 15,171 |

Table S3 Alpha diversity indexes of environmental samples. *P* value is the significance of t test between control and GO treatment group.

| Sample | Group | Bacterial community | | | Fungal community | | |
| --- | --- | --- | --- | --- | --- | --- | --- |
|  |  | Richness | Shannon | PD | Richness | Shannon | PD |
| A1 | Control | 3,079 | 6.40 | 153.42 | 529 | 3.92 | 153.42 |
| A3 |  | 4,328 | 6.57 | 206.25 | 573 | 4.01 | 206.25 |
| E1 |  | 4,686 | 6.87 | 226.61 | 643 | 4.12 | 226.61 |
| E3 |  | 4,201 | 6.47 | 201.10 | 618 | 4.18 | 201.10 |
| F1 |  | 3,962 | 6.65 | 186.30 | 412 | 3.41 | 186.30 |
| F3 |  | 3,788 | 6.69 | 173.99 | 320 | 2.31 | 77.27 |
| H1 |  | 3,539 | 6.39 | 165.49 | 423 | 3.70 | 83.13 |
| H3 |  | 3,280 | 6.38 | 149.16 | 350 | 3.67 | 91.54 |
| L1 |  | 4,122 | 7.07 | 192.83 | 398 | 3.55 | 87.58 |
| L3 |  | 3,273 | 6.65 | 144.57 | 201 | 2.59 | 69.97 |
| M1 |  | 3,764 | 6.82 | 179.23 | 376 | 3.75 | 39.83 |
| M2 |  | 3,408 | 6.56 | 153.32 | 313 | 3.77 | 70.47 |
| A1_GO | GO treatment | 3,530 | 6.36 | 166.20 | 178 | 1.91 | 18.49 |
| A3_GO |  | 3,745 | 6.43 | 165.89 | 197 | 1.78 | 20.09 |
| E1_GO |  | 3,891 | 6.49 | 175.21 | 233 | 1.87 | 30.36 |
| E3_GO |  | 3,709 | 6.29 | 170.29 | 265 | 1.69 | 37.05 |
| F1_GO |  | 4,365 | 6.91 | 208.63 | 569 | 3.51 | 84.42 |
| F3_GO |  | 3,983 | 6.92 | 180.06 | 440 | 4.83 | 23.65 |
| H1_GO |  | 3,735 | 6.74 | 170.60 | 384 | 2.93 | 62.77 |
| H3_GO |  | 3,511 | 6.55 | 161.27 | 276 | 3.46 | 47.03 |
| L1_GO |  | 3,973 | 6.92 | 181.64 | 350 | 3.34 | 56.40 |
| L3_GO |  | 3,148 | 6.57 | 136.58 | 505 | 3.83 | 66.52 |
| M1_GO |  | 3,363 | 6.65 | 153.24 | 321 | 3.52 | 54.90 |
| M2_GO |  | 3,270 | 6.48 | 147.52 | 611 | 3.48 | 86.82 |
| *P* value | Control vs GO treatment | 0.408 | 0.781 | 0.178 | 0.375 | 0.218 | 0.094 |
